# A Comparative Study of Structural Representations for 2D Materials: Insights from Dynamic Collision Fingerprint and Matminer Library

**DOI:** 10.1021/acsomega.6c03154

**Published:** 2026-07-02

**Authors:** Raphael M. Tromer, Isaac M. Felix, Rafael Besse, Marcelo L. Pereira Junior, Marcos G. E. da Luz

**Affiliations:** † Institute of Physics, University of Brasília, 70910-900 Brasília, Federal District, Brazil; ‡ Center for Agri-food Science and Technology, 154624 Federal University of Campina Grande, 58840-000 Pombal, Paraíba, Brazil; § International Center of Physics, Institute of Physics, 564113 University of Brasília, 70910-900 Brasília, Federal District, Brazil; ∥ Department of Electrical Engineering, College of Technology, University of Brasília, 70910-900 Brasília, Federal District, Brazil; ⊥ Department of Physics and Multidisciplinary Laboratory for Modeling and Analysis of Data in Complex Systems (MADComplex), Center for Scientific Modeling and Computing, 28122 Federal University of Paraná, 81531-980 Curitiba, Paraná, Brazil

## Abstract

In materials science, the choice of structural descriptors for machine learning protocols strongly influences both predictive performance and model interpretability. High-dimensional descriptors can improve numerical accuracy, but often introduce substantial computational overhead and reduce transparency. To address this, the Dynamic Collision Fingerprint (DCF) framework generates concise descriptors via the dynamical probing of atomic structures. In this work, we benchmark DCF against the widely used Matminer library using a data set of 120 two-dimensional (2D) carbon allotropes. We evaluate performance across three regression algorithms, linear regression, decision trees, and XGBoost, utilizing train-test partitions from 10% to 90%. Our results demonstrate that DCF matches the predictive accuracy of Matminer across all algorithms. Although Matminer can be faster in terms of execution time, DCF accomplishes its predictive performance using descriptors that are significantly lower-dimensional, pointing to manageable computing costs in feature space. Moreover, compared to the rather technical Matminer descriptions, DCF exhibits considerably clearer physical interpretability. These findings suggest that DCF serves as a viable alternative to high-dimensional descriptor libraries for structural representation, since it remains both computationally flexible and physically grounded.

## Graphical abstract

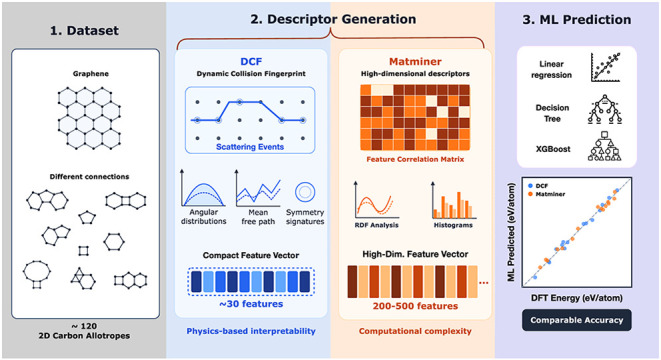

## Introduction

Atomic level structural characterization constitutes a central element of materials science and computational chemistry. It provides the basis for establishing structure–property relationships that guide both experimental interpretation and the rational design of novel materials.^1–9^ Over the past decade, the integration of atomistic simulations, structural descriptors, and machine learning models has significantly transformed high throughput materials discovery.^8–13^ In this context, the definition of descriptors that are simultaneously efficient, interpretable, and transferable has emerged as a critical challenge, since the predictive capability and robustness of machine learning models depend strongly on the quality and representational adequacy of the selected structural features.^14–16^

A large variety of structural descriptors has been proposed and consolidated in the literature.^17–24^ These approaches include representations based on radial and angular distribution functions, orientational order parameters, spectral formulations such as the Smooth Overlap of Atomic Positions (SOAP) method,^17^ and graph based or topology driven embeddings derived from message passing frameworks.^24,25^ Many of these descriptors are available through dedicated software libraries. Among them Matminer^26^ has become widely adopted in computational materials modeling due to its extensive collection of structural, electronic, and chemical features and its support for reproducible workflows across diverse material classes.

Despite such versatility, the employment of generic high-dimensional descriptor libraries often introduces practical limitations when applied to structurally complex systems, the case of two-dimensional (2D) materials. A first limitation concerns the physical interpretability of many features, as they cannot be directly associated with intuitive structural characteristics. A second limitation concerns sensitivity to disorder, defects, and aperiodicity, which are frequently present in 2D systems, where local distortions, vacancies, and topological irregularities are common.^27–30^ Beyond idealized atomically thin crystals, the broader class of complex 2D materials also includes confined and interfacially stabilized systems, such as the graphitic-like gallium nitride obtained at the graphene/SiC interface^31^ and the 2D indium oxide proposed at the same interface,^32^ whose structural diversity further requires the development of robust and physically meaningful descriptors for data-driven analysis.

Motivated by these challenges, a recently proposed descriptor scheme, the Dynamic Collision Fingerprint (DCF), was introduced aiming to be a more physically based alternative.^33^ Guided by well-established concepts from classical statistical mechanics,^34,35^ DCF departs from static coordinate-based representations. Instead, it seeks to dynamically probe atomic structures through trajectories of idealized particles undergoing elastic collisions with the lattice. In this way, statistical analyses of free paths, collision angles, recurrence events, and angular symmetries obtained through Fourier analysis and Shannon entropy allow the descriptor to encode structural signatures associated with symmetry, porosity, and disorder.^33^ This construction yields descriptors that are directly interpretable in physical terms, independent of electronic or energetic models, and computationally adjustable according to the assumed sampling parameters.

Notwithstanding the promising performance of DCF displayed in the original work,^33^ a thorough evaluation against popular high-dimensional descriptor libraries and under controlled and statistically consistent benchmarking settings is still missing. In this contribution we fulfill this gap by systematically contrasting the DCF with the Matminer descriptor library. Using a data set composed of 120 distinct 2D carbon allotropes, descriptors generated by both approaches are considered within three representative machine learning models, namely, linear regression,^36^ decision trees,^37^ and XGBoost.^38^ Also, a wide variety of train and test partitions are examined in the comparisons.

The comprehensive analyses carried out in the present work allow for the evaluation of DCF’s relative performance in terms of physical interpretability, descriptor compactness, computational cost-performance trade-off, prediction accuracy, and resilience. Indeed, our results demonstrate that DCF can serve as a complementary and, in certain settings, competitive descriptor framework for materials informatics workflows. Specifically, by framing structural characterization as a dynamical response problem rather than a static geometric structure, we demonstrate that predictors can remain remarkably compact while retaining the critical information required to accurately model 2D materials, even though the baseline descriptor generation cost may depend on the sampling settings.

## Methodology

The methodology followed here comprises data set preparation, descriptor construction, machine learning modeling, and statistical evaluation, all performed under consistently specified conditions to ensure direct comparability between descriptor approaches. A schematic overview of the complete workflow adopted in this study is presented in Figure 1, summarizing the main stages from data set preparation to descriptor generation, model training, and statistical validation.

**Figure 1:**
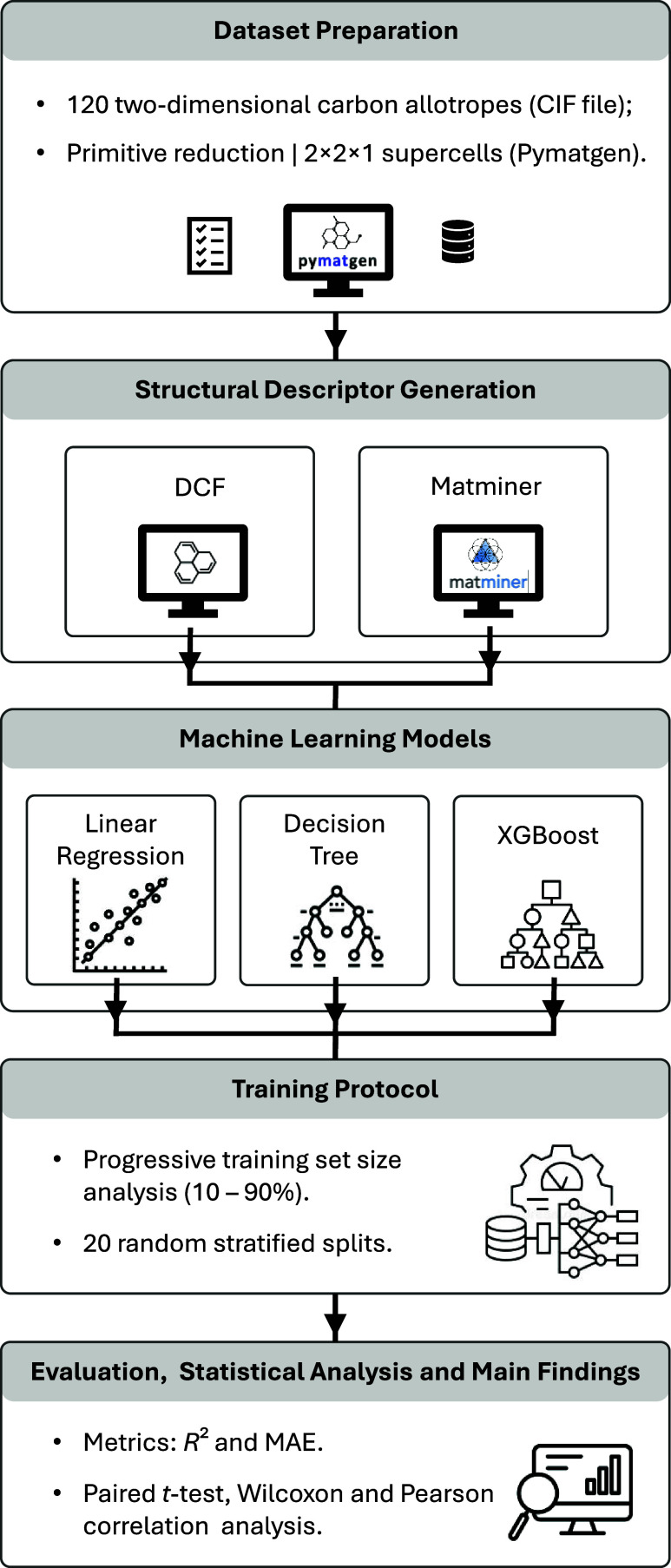
Schematic workflow of the computational pipeline employed in this study. After data set preparation, structural descriptors were generated using DCF and Matminer, and the models were trained using linear regression, decision trees, and XGBoost. Performance was assessed through MAE and *R* ^2^, complemented by paired statistical tests and correlation analyses.

The benchmark data set employed in this study is entirely computational, comprising 120 distinct 2D carbon allotropes whose structures and formation energies were obtained from density functional theory (DFT) calculations reported in the literature.^39^ Although materials databases can in general be assembled from either first-principles calculations or experimental measurements, the benchmark adopted in this work is specifically based on DFT-derived data. All coordinates and lattice information for the addressed structures are provided in the Supporting Information (SI). The systems were standardized using the Pymatgen library.^40^ Each structure was reduced to its primitive cell, assuming a symmetry tolerance of 10^–3^ Å, and atomic coordinates were scaled to ensure uniform density normalization. The chosen target property is the formation energy reported in the literature.^39^ Then, supercells of at least 2 × 2 × 1 are required for appropriate simulations, and this was consistently adopted throughout the study.^33^

Two descriptor frameworks were considered, DCF and the Matminer feature library. The DCF model is explained in detail in,^33^ but its essential points can be summarized as follows. One considers the propagation of classical point particles undergoing elastic collisions within an atomic lattice supercell under periodic boundary conditions. Each trajectory propagates during a specific number of steps *N*_S_. A single “step” corresponds to a straight line segment between successive boundary crossings. For proper averages, a total of *N*_L_ trajectories are launched. Along propagation, quantities such as traveled distances and angular deflections are recorded. The final descriptor vector is constructed from statistical analyses of angular distributions using Shannon entropy as well as Fourier decomposition of recurrence frequencies (associated with one-to-9-fold lattice symmetries), mean free paths, and recurrence times, with explicit expressions provided in.^33^

Unless otherwise explicitly stated, the standard parametrization was *N*_S_ = 10^4^ and *N*_L_ = 200, yielding descriptor vectors from 25 to 30 dimensions. Larger *N*_S_ and *N*_L_ were considered for sensitivity analysis, with the fast (extended) configuration corresponding to *N*_S_ = 10^3^ and *N*_L_ = 100 (*N*_S_ = 10^4^ and *N*_L_ = 300). The implementation relies on NumPy, SciPy, and pandas and is available in the repository indicated in this work.

Matminer descriptors were generated using the Matminer library,^26^ combining three featurizers, namely density features, the radial distribution function, and bond fractions. The structures were read from the corresponding CIF files using Pymatgen. The density descriptor was used in its default configuration, yielding the mass density, the volume per atom, and the packing fraction. The radial distribution function was discretized with a cutoff of 20.0 Å and a bin size of 0.1 Å, generating 200 binned components. Bond fractions were computed using CrystalNN as the neighbor-detection algorithm, with exact rather than approximate bond fractions and no prespecified restriction on allowed bond types. Because the bond-fraction descriptor requires the set of allowed bond types to be defined prior to feature extraction, it was fitted to the full structure set before descriptor computation. Depending on the bond-fraction contribution, which is determined by the chemistry of the data set, the resulting feature vectors contain approximately 200–500 components, thus substantially larger than those of DCF. All descriptors were normalized using *z*-score scaling prior to training. For a full Matminer featurized specification see Section S1 of the SI.

Three representative regression algorithms were addressed. Linear regression was implemented using ordinary least-squares from scikit learn. The decision tree model was trained with a maximum depth of 8 and a minimum sample count per leaf of 2. The XGBoost model was implemented with the hyperparameters n_estimators, max_depth, learning_rate, and subsample set to 500, 8, 0.05, and 0.8, respectively. Prior to training, all features were standardized to a mean of 0 and a variance of 1. No feature selection or dimensionality reduction was applied in order to preserve direct comparability between descriptor schemes. Fixed hyperparameters were adopted for all models, as the objective here is to compare descriptor families under identical modeling conditions rather than to optimize individual performance. But to verify that this descriptor-level comparison is not biased by the common fixed-hyperparameter setup, an independent hyperparameter optimization based on Optuna was additionally carried out for the XGBoost regressor and for both descriptor families. The full protocol, the search space, and the resulting performance comparison are reported in Section S2 of the SI.

Predictive robustness was assessed through repeated random train and test partitions, where the test fraction *X*_T_ denotes the portion of the data set assigned to the test set. We considered *X*_T_ values ranging from 0.1 to 0.9 in increments of 0.1. Each partition was repeated 20 times with random seeds to ensure statistical reliability, and reported quantities correspond to averages across all seeds. To make the statistical variability across the 20 random train and test partitions explicit, the XGBoost results are additionally reported with error bars in Section S3 of the SI, for both DCF and Matminer and for both the baseline and the Optuna optimized configurations. These extra results confirm that the discussed trends remain within the corresponding uncertainty ranges, while also making transparent the larger dispersion observed at high test fractions, particularly for *R* ^2^, as expected for a data set of limited size.

Predictive performance was quantified using the coefficient of determination *R* ^2^ and the mean absolute error (MAE), where MAE corresponds to the average absolute difference between predicted and reference values. To evaluate whether DCF and Matminer exhibit statistically distinguishable performance and to quantify their concordance across test fractions, paired *t* tests, Wilcoxon signed rank tests, and Pearson correlation analyses were performed on the resulting metric distributions. Statistical analyses were carried out using SciPy, adopting a significance threshold of *p* < 0.05.

## Results and Discussion

The predictive performance of DCF descriptors was first evaluated with respect to the simulation parameters controlling trajectory sampling. Figure 2 summarizes the dependence of the MAE on *X*_T_ for different combinations of *N*_S_ and *N*_L_ and for the three machine learning models considered in this work.

**Figure 2:**
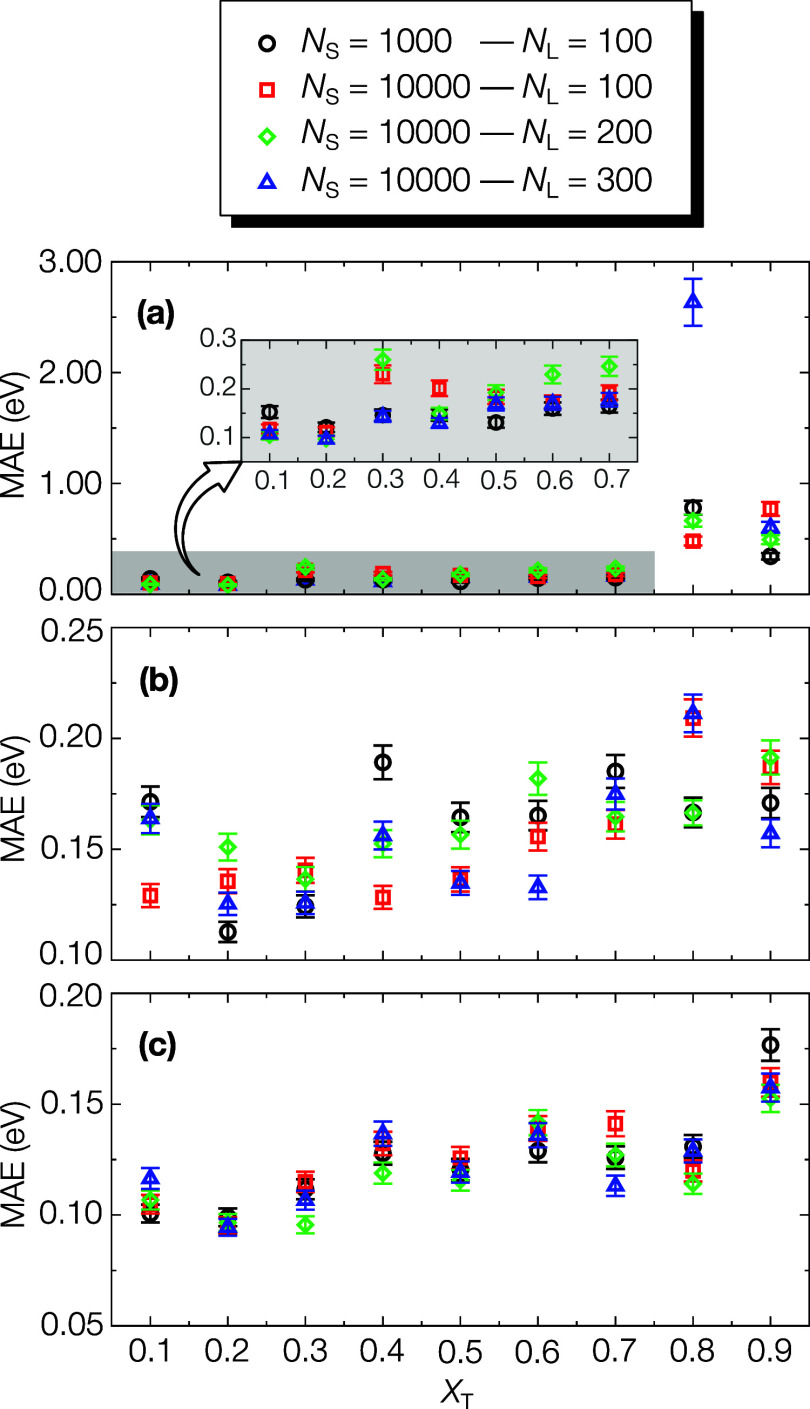
MAE as a function of *X*_T_ for different DCF parametrizations defined by *N*_S_ and *N*_L_ for (a) linear regression, (b) decision tree, and (c) XGBoost.

Across all models, the MAE remains stable with respect to variations in *N*_S_ and *N*_L_. As observed in Figure 2(a), the only noticeable deviation corresponds to a single outlier near *X*_T_ ≈ 0.8 in the linear regression case. In contrast, Figure 2(b),2(c) show tightly grouped results across the full range of *X*_T_, with differences comparable to the expected statistical fluctuations from random train-test partitions. This behavior indicates that the DCF descriptors are robust with respect to moderate variations in trajectory length and sampling density, and that convergence is achieved without the need for extensive parameter tuning.

This stability aligns with the central concept of the DCF formulation:^33^ structural information should be better captured through aggregated dynamical statistics rather than through static high-dimensional signatures. Once the trajectory sampling explores the relevant structural motifs, the resulting descriptors become statistically stable. In the present data set, increasing *N*_L_ beyond 100 does not produce systematic gains in predictive accuracy, indicating that relatively inexpensive parametrizations are already sufficient to capture the dominant structural information required for machine learning predictions.

For the following analyses, we set *N*_S_ = 10^4^ and *N*_L_ = 200, representing a balanced trade-off between statistical stability and computational cost. Figure 3 compares the predictive performance obtained with DCF and Matminer descriptors through the MAE as a function of *X*_T_ for our three machine learning models. The results illustrate that DCF systematically reaches predictive errors comparable to those obtained with Matminer, despite its substantially lower dimensionality and simpler construction.

**Figure 3:**
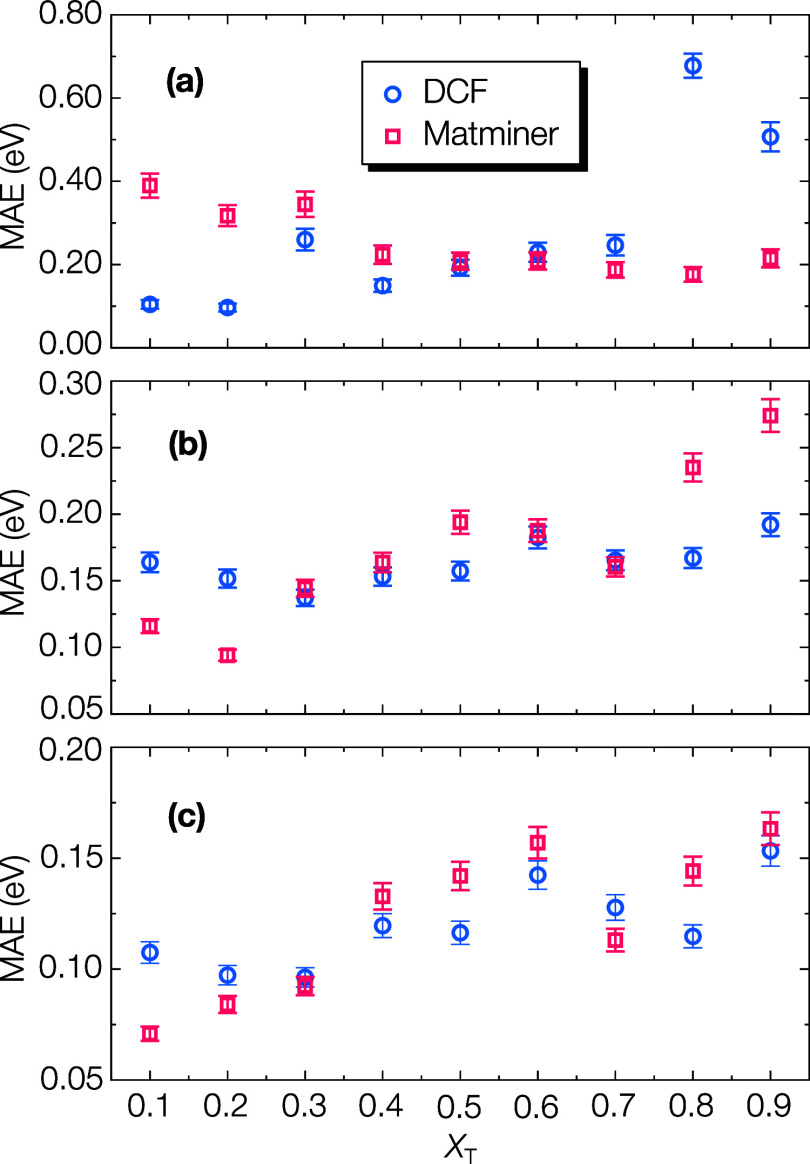
MAE as a function of *X*_T_ comparing DCF and Matminer descriptors for (a) linear regression, (b) decision tree, and (c) XGBoost.

For linear regression, depicted in Figure 3(a), DCF frequently produces similar or slightly smaller MAE values across several test fractions, with more pronounced differences emerging at larger *X*_T_, where the reduced amount of training data amplifies sensitivity to descriptor quality. In this regime, both approaches occasionally exhibit larger fluctuations, which is expected given the limited expressive capacity of linear models for complex structure–property relationships. For decision trees, Figure 3(b) shows that the two descriptor frameworks lead to closely overlapping MAE values across most test fractions. Also, both representations provide similar levels of structural information for moderate nonlinear learning.

**Figure 4:**
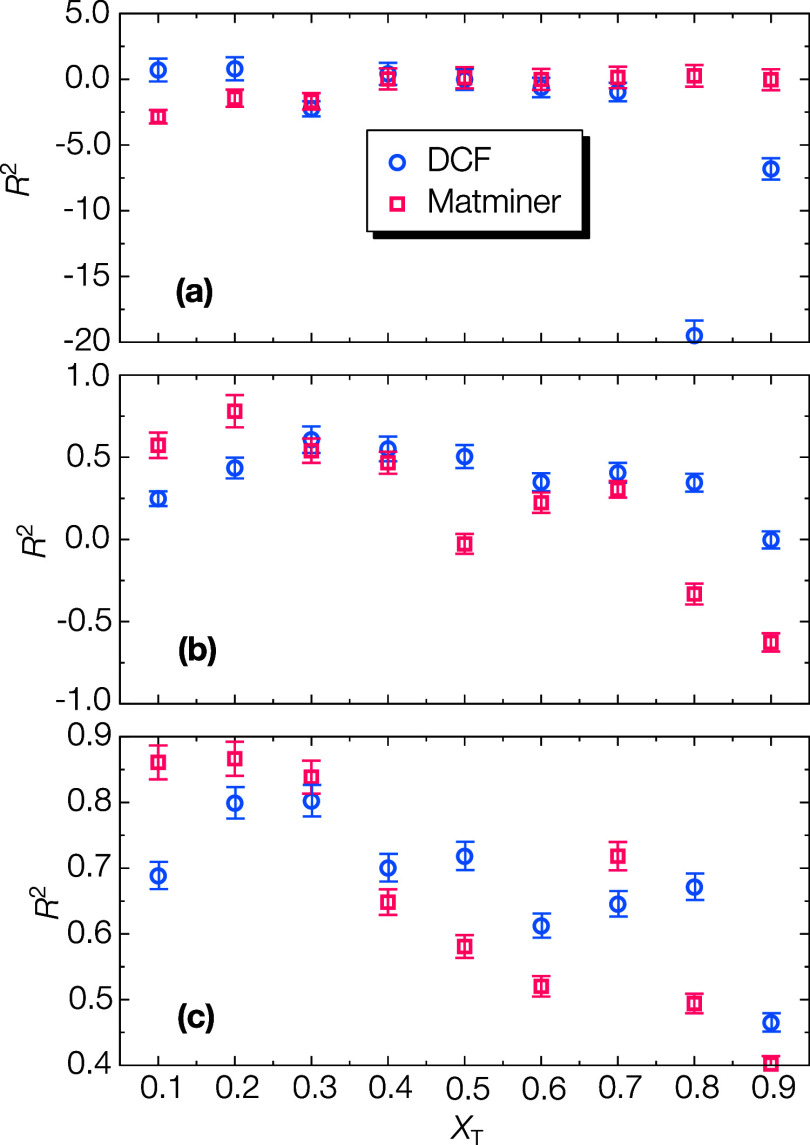
*R* ^2^ as a function of *X*_T_ comparing DCF and Matminer descriptors for (a) linear regression, (b) decision tree, and (c) XGBoost.

The comparison becomes even more consistent for XGBoost, as seen from Figure 3(c), which reveals nearly indistinguishable results between DCF and Matminer over the entire range of *X*_T_. This indicates that the essential predictive information captured by the higher-dimensional Matminer representation is effectively retained within the compact DCF when coupled to a strong nonlinear learner.

An additional perspective on the predictive capability discussed above is obtained by analyzing the coefficient of determination, whose evolution *X*_T_ is shown in Figure 4 for DCF and Matminer descriptors. We recall that while MAE quantifies the absolute prediction error, it *R* ^2^ provides additional insight into how effectively each descriptor framework captures the variance of the target property across different train and test partitions.

In the linear regression case, Figure 4(a), both descriptor sets lead to low predictive power, with *R* ^2^ values fluctuating around zero and occasionally becoming strongly negative at larger *X*_T_. This trait is consistent with the limited flexibility of linear models when applied to complex, intrinsically nonlinear structure–property relationships, reinforcing the interpretation already suggested by the MAE analysis.The strongly negative *R* ^2^ values observed in the most unfavorable regimes, particularly for the linear model at large *X*_T_, do not indicate a flaw in the evaluation procedure but rather reflect the well-known sensitivity of *R* ^2^ under small training sample conditions, where the prediction error can exceed the variance of the corresponding test sample. We should note that the interpretability advantage attributed here to DCF is intended at the descriptor-definition level and is further illustrated in Section S4 of the SI for graphene as a reference structure. Whereas the Matminer representation is dominated by a large set of distance-binned components together with a few auxiliary scalar quantities, the DCF descriptor is composed of compact variables with direct physical meaning, including free-path statistics, relaxation-time-related quantities, diffusivity, angular entropy, and symmetry-resolved intensities. The 6-fold component provides an immediate descriptor-level link to the hexagonal symmetry of graphene. Moreover, the decision tree model, Figure 4(b), indicates a clear gain in predictive power, with predominantly positive *R* ^2^ values across the tested splits. The agreement between DCF and Matminer remains strong, for the small differences observed in specific regions do *X*_T_ not alter the overall picture of comparable performance.

The aforementioned agreement becomes even stronger for XGBoost, Figure 4(c). Indeed, the plots show consistently high *R* ^2^ values for both descriptor frameworks. The close overlap between the results confirms that DCF captures essentially the same predictive information content as Matminer when combined with a sufficiently expressive nonlinear learner. Variations between individual points remain within the range expected from statistical fluctuations across different random train-test partitions.

Beyond just the crude face value of predictive metrics, it is also relevant to examine how the two descriptor protocols differ in terms of dimensionality, interpretability, and computational cost, as these aspects directly affect their practical applicability in large-scale materials informatics workflows. A central distinction concerns feature dimensionality and physical transparency. The DCF representation remains compact, typically containing approximately 25 to 30 descriptors, each one directly associated with a physical or geometric characteristic, as the already mentioned mean free path, recurrence time, angular entropy, and rotational symmetry intensities from 1 to 9-fold. In contrast, the Matminer representation contains several hundred features, largely derived from discretizing the radial distribution function across multiple distance bins (up to approximately 20 Å), complemented by density and packing statistics. While such a high-dimensional description is versatile and broadly applicable, individual components are less intuitive from a physical perspective, noting that isolated RDF bins rarely carry a clear standalone interpretation.

A second relevant aspect concerns computational cost. In the current workflow, Matminer requires approximately 10 s per structure, whereas DCF in its standard configuration (*N*_S_ = 10^4^, *N*_L_ = 200) requires about 4 min per structure. Therefore, the practical utility of DCF does not stem from a uniformly lower raw generation cost compared to Matminer in standard settings. Rather, its primary advantage lies in combining descriptor compactness, physical interpretability, and competitive predictive performance, alongside a tunable cost-accuracy trade-off. In this context, the fast configuration (*N*_S_ = 10^3^, *N*_L_ = 100) becomes particularly relevant. It yields MAE values remarkably close to those of extensive sampling while reducing the average wall time to roughly 30 s per structure, bringing the computational footprint to a comparable order of magnitude. Accordingly, the practical relevance of DCF in this benchmark stems less from a lower raw generation time than from its compact, physically interpretable representation and the ability to reduce computational overhead through relaxed sampling parameters without altering the descriptor-level conclusions.

Combined with the results presented in Figures 3 and 4, these observations highlight a clear trade-off between descriptor compactness, interpretability, and computational cost. Linear models remain limited across both descriptor frameworks, whereas decision tree and XGBoost models consistently achieve high predictive performance, demonstrating that DCF preserves the essential structural information despite its reduced dimensionality. A consolidated comparison summarizing dimensionality, interpretability, computational cost, and predictive behavior is presented in Table 1. To confirm that this descriptor-level comparison is not an artifact of the common fixed-hyperparameter setup, an independent Optuna-based hyperparameter optimization was performed for XGBoost on both DCF and Matminer, with the complete protocol reported in Section S2 of the SI. The optimized results introduce only minor quantitative variations and do not modify the overall conclusion that the two descriptor families display closely comparable predictive performance. The interpretability advantage attributed here to DCF is intended at the descriptor-definition level and is further illustrated in Section S4 of the SI for graphene as a reference structure. Whereas the Matminer representation is dominated by a large set of distance-binned components together with a few auxiliary scalar quantities, the DCF descriptor is composed of compact variables with direct physical meaning, including free-path statistics, relaxation-time-related quantities, diffusivity, angular entropy, and symmetry-resolved intensities. The 6-fold component provides an immediate descriptor-level link to the hexagonal symmetry of graphene.

**Table 1:** Comparison between DCF and Matminer in Terms of Feature Dimensionality, Interpretability, Descriptor-Generation Cost Per Structure, and Overall Predictive Behavior^a^

descriptor set	#features	interpretability	time per structure	performance summary
DCF (Standard)	∼25–30	High (physics based)	∼4 min	Predictive accuracy comparable to Matminer for both MAE and *R* ^2^, with consistent performance for decision tree and XGBoost models.
DCF (Fast)	∼25–30	High (physics based)	∼30 s	Results remain very close to the standard configuration, with differences within the statistical variations observed in Figure 2.
Matminer	∼200–500	Moderate to low (bin based)	∼10 s	Comparable MAE and *R* ^2^ behavior relative to DCF, showing similar trends for decision tree and XGBoost models.

a DCF standard corresponds to **N**_S_ = 10^4^ and **N**_L_ = 200, while DCF fast corresponds to **N**_S_ = 10^3^ and **N**_L_ = 100. Performance summaries are consistent with the trends discussed from Figures 3 and 4. For the Matminer descriptor set, the dimensionality range depends on the selected presets, including RDF binning range and resolution, as well as optional feature subsets.

To further complement the performance trends discussed above, additional statistical analyses were conducted to evaluate whether DCF and Matminer exhibit significant differences across the full ensemble of repeated random splits. Paired statistical tests were applied to the metric distributions obtained from 20 random seeds for each *X*_T_ value, along with correlation analyses across test fractions. This provides a quantitative assessment of agreement between descriptor frameworks beyond direct visual comparison.

For linear regression, no statistically significant differences were detected between DCF and Matminer for either *R* ^2^ or MAE. Both paired *t*-tests and Wilcoxon signed-rank tests yielded nonsignificant outcomes (*p* > 0.05), indicating that the small variations observed between descriptors are compatible with statistical fluctuations rather than systematic performance differences. Correlation analyses across test fractions revealed weaker alignment than in nonlinear models, consistent with the higher variability observed in this regime, particularly at large *X*_T_’s, when both descriptor sets can produce unstable, even negative, *R* ^2^ values. For the decision tree model, paired statistical tests again indicated no relevant distinctions between DCF and Matminer for either MAE or *R* ^2^ (*p* > 0.05). Also, performance trends across test fractions remain positively correlated, pointing to similar responses of both descriptor frameworks to variations in training-set size. Regimes where performance improves or degrades, therefore, tend to coincide for DCF and Matminer, corroborating the traits observed in Figures 3(b) and 4(b). For XGBoost, paired *t*-tests and Wilcoxon tests show that DCF and Matminer are statistically indistinguishable across all evaluated metrics (*p* > 0.05), while correlation analyses across test fractions reveal strongly positive alignment for both MAE and *R* ^2^.

## Conclusions

This work presented a systematic comparison of the DCF and Matminer descriptor frameworks, evaluating their predictive performance across multiple machine learning models and train and test data splits. The analysis combined direct performance curves, statistical tests, and an explicit comparison of descriptor dimensionality, interpretability, and computational cost.

Across the full set of simulations, both descriptors exhibited similar predictive limits under linear regression, in which model simplicity dominates the learning behavior and leads to unstable performance at large test fractions. In contrast, nonlinear learners consistently displayed higher accuracy, as it should be expected given the data set inherently nonlinear structure–property relationships. Notably, for decision tree and XGBoost models, DCF and Matminer converged to closely aligned performance profiles. Statistical tests confirmed the absence of significant differences between the two descriptor sets, while correlation analyses across test fractions revealed consistent predictive trends.

More generally, the present contribution comprehensively illustrates a key trade-off between descriptor compactness, interpretability, and computational cost. On one hand, Matminer offers a broad and computationally inexpensive representation but relies on a high-dimensional feature space that is less directly interpretable. On the other hand, the stability of DCF under reduced sampling parameters enables substantial reductions in computational overhead without significant loss of accuracy, reinforcing its practical relevance as a tunable framework rather than a strictly cheaper alternative. These features support the use of DCF as a physically grounded, compact approach for data-driven investigations of structure–property relationships in complex materials, particularly when model interpretability (as concretely illustrated in the Section S4 of the SI) and flexible sampling are of interest.

## Supplementary Material



## Notes

Detailed Matminer featurizer specification (Section S1); Optuna-based hyperparameter optimization protocol, search space, and comparison against the fixed-hyperparameter baseline for XGBoost on both descriptor families (Section S2, Figures S1 and S2); statistical uncertainty across the 20 random train and test partitions for XGBoost, with and without Optuna optimization (Section S3 and Figure S3); descriptor-level interpretability example for graphene comparing Matminer and DCF representations (Section S4 and Figure S4); coordinates and lattice information for the 120 two-dimensional carbon allotropes used in the benchmark (PDF)
