# Supplementary material for: A Comparative Study of Structural Representations for 2D Materials: Insights from Dynamic Collision Fingerprint and Matminer Library
Source: ACS Omega. 2026 Jul 2;11(28):42389–96. doi: 10.1021/acsomega.6c03154 (PMC13393029; doi:10.1021/acsomega.6c03154)
Supplement: Supplementary file 1 [file ao6c03154_si_001.pdf]

Supporting Information: A Comparative Study of Structural Representations for 2D Materials: Insights from Dynamic Collision Fingerprint and Matminer Library

Raphael M. Tromer,[†] Isaac M. Felix,[‡] Rafael Besse,[¶] Marcelo L. Pereira Junior,[*,§] and Marcos G. E. da Luz[‖]

†Institute of Physics, University of Brasília, 70910-900 Brasília, Federal District, Brazil

‡Center for Agri-food Science and Technology, Federal University of Campina Grande, 58840-000 Pombal, Paraíba, Brazil

¶International Center of Physics, Institute of Physics, University of Brasília, 70910-900 Brasília, Federal District, Brazil

§Department of Electrical Engineering, College of Technology, University of Brasília, 70910-900 Brasília, Federal District, Brazil

‖Department of Physics and Multidisciplinary Laboratory for Modeling and Analysis of Data in Complex Systems (MADComplex), Center for Scientific Modeling and Computing, Federal University of Paraná, 81531-980 Curitiba, Paraná, Brazil

E-mail: marcelo.lopes@unb.br

This document provides additional methodological detail and complementary analyses supporting the main manuscript. Section specifies the exact Matminer featurizer composition employed in this work. Section describes the hyperparameter optimization protocol based on Optuna and reports the corresponding performance comparison against the fixed-hyperparameter baseline. Section presents the statistical dispersion across repeated random train and test partitions for the XGBoost model. Section illustrates the descriptor-level interpretability of DCF using graphene as a reference structure.

S1. Matminer featurizer specification

For full reproducibility of the comparison reported in the main manuscript, the Matminer descriptor vector was assembled from three structural featurizers of the Matminer library, combining density features, the radial distribution function, and bond fractions. The crystallographic input was read from the corresponding CIF files using Pymatgen.

The density descriptor was used in its default configuration, yielding three scalar quantities, namely the mass density, the volume per atom, and the packing fraction. The radial distribution function was discretized with a cutoff of 20.0 Å and a bin size of 0.1 Å, yielding 200 binned components representing the pair correlation up to the cutoff distance. Bond fractions were computed using CrystalNN as the neighbor-detection algorithm, with exact rather than approximate bond fractions and no pre-specified restriction on allowed bond types. Because the bond-fraction descriptor requires the set of allowed bond types to be defined prior to feature extraction, it was fitted to the full structure set before computing the individual sample descriptors. All resulting features were subsequently standardized via z-score normalization to zero mean and unit variance across the dataset before being passed to the regression models.

S2. Hyperparameter optimization with Optuna

To address the concern that the descriptor-level comparison reported in the main manuscript could be affected by the choice of fixed model hyperparameters, an independent hyperparameter optimization study was conducted for the XGBoost regressor, separately for the DCF and Matminer descriptor sets. XGBoost was selected because it is the most expressive nonlinear learner considered in this work and therefore the most sensitive to model tuning. The same XGBoost hyperparameters that determine the regressor's capacity were passed to the optimizer, while the remaining configuration was held constant to ensure reproducibility. Specifically, histogram-based tree building was used, the training objective was squared-error regression, RMSE served as the evaluation criterion for early stopping, and early stopping was applied with a patience of 50 rounds on the internal validation holdout. A fixed random seed ensured deterministic results across runs.

The optimization was carried out with the Optuna framework, using a total of 60 trials per execution. For each value of the test fraction X_{T} from 0.1 to 0.9 and for each descriptor family, Optuna was run independently. Within each Optuna run, the optimizer only had access to the training portion of the data. The test set was held out and was never inspected during the search. The objective function was the root mean square error evaluated on an internal training-validation holdout set, and the search proceeded by minimizing this internal validation error. No k-fold cross-validation was used within the inner loop. After the 60 trials concluded, the best configuration was retrained on the full training portion and evaluated on the previously untouched test set, providing the optimized performance values reported below. The complete search space is summarized in Table S1.

The comparison between the optimized and the fixed hyperparameter results is presented in Figs. S1 and S2 for the Matminer and the DCF descriptor sets, respectively. The top row of each figure reports the training values of R^2, RMSE, MSE, and MAE as a function of X_{T}, while the middle row reports the corresponding test values. The bottom row reports the difference between the optimized and the baseline configurations, defined as $\Delta = (\mathrm{Optuna}) -$

Table S1: Search space of the Optuna optimization for XGBoost. Logarithmic ranges are indicated explicitly.

Hyperparameter	Range	Scale
`n_estimators`	200 to 3000	linear
`learning_rate`	10^{-3} to 0.2	logarithmic
`max_depth`	3 to 12	linear
`min_child_weight`	1 to 15	linear
`subsample`	0.5 to 1.0	linear
`colsample_bytree`	0.5 to 1.0	linear
`gamma`	0.0 to 10.0	linear
`reg_lambda`	10^{-8} to 50.0	logarithmic
`reg_alpha`	10^{-8} to 50.0	logarithmic
`max_delta_step`	0.0 to 10.0	linear

(baseline), so that a negative Δ for R^2 and a positive Δ for an error metric correspond to a degradation upon optimization.

Across the full range of X_{T} examined, the differences between the optimized and the baseline configurations remain small and oscillate around zero, with no systematic sign that would indicate a uniform improvement upon optimization. The most pronounced deviations are observed at $X_{\mathrm{T}} = 0.9$, which corresponds to the regime in which only roughly twelve structures remain in the training set. In this regime, the internal validation holdout used by Optuna becomes statistically unreliable, and the optimized model occasionally overfits to a small sample, resulting in degraded test performance. This behavior is consistent with expectations for hyperparameter search on small training sets and does not reflect a property of the descriptor families themselves. Most importantly, the relative ranking between DCF and Matminer is preserved after optimization. The descriptor-level conclusions of the main manuscript, namely that DCF and Matminer achieve comparable predictive accuracy under XGBoost, therefore do not depend on the specific choice of fixed hyperparameters used in the baseline analysis.

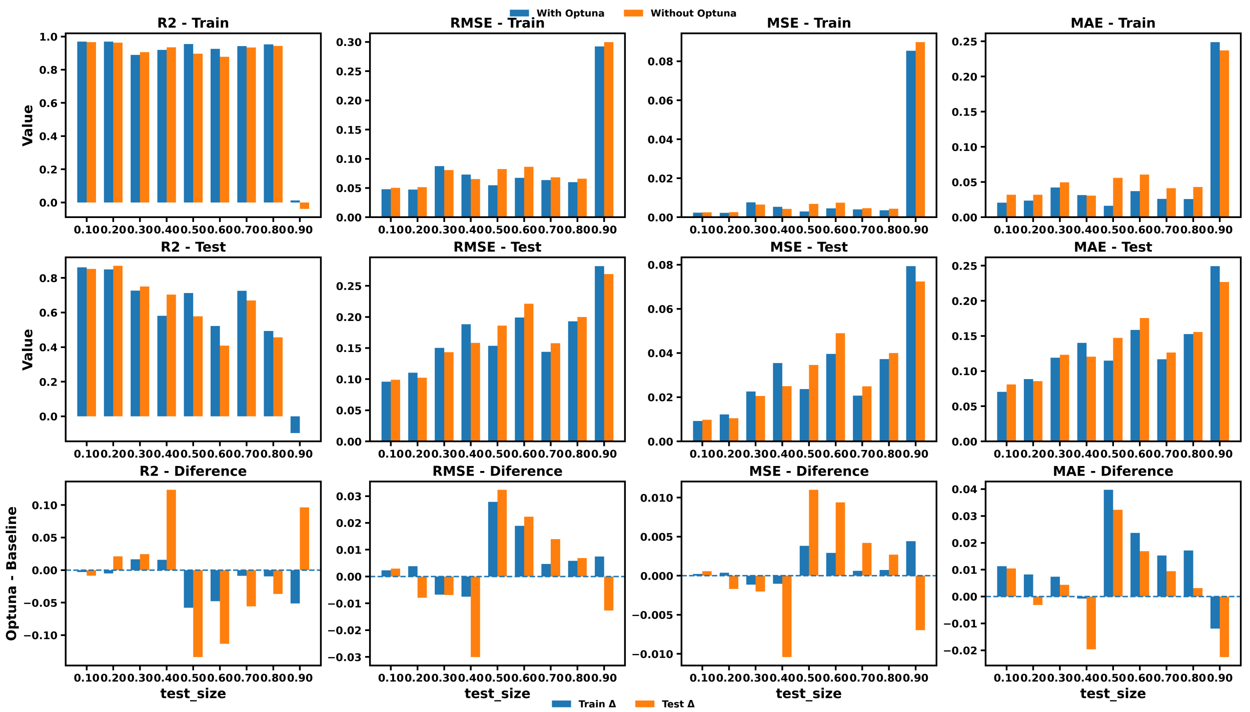

Figure S1: S3. XGBoost performance with and without Optuna-based hyperparameter optimization for the Matminer descriptor set. Top row, training values of R^2, RMSE, MSE, and MAE as a function of X_{T}. Middle row, corresponding test values. Bottom row, optimized minus baseline differences for training (blue) and test (orange).

Statistical uncertainty across repeated random splits

The benchmark dataset contains only 120 structures, so the choice of a particular random train-test partition can introduce non-negligible variability in the metric values reported in the main manuscript. To make this variability explicit, the XGBoost results were reanalyzed for both descriptor families, with and without Optuna-based hyperparameter optimization, using the same protocol of 20 random seeds per value of X_{T}. For each seed, the model was trained on the corresponding training portion and evaluated on the held-out test portion. The reported quantities are the mean and the standard deviation of the resulting metric values across the 20 seeds.

Figure S3 reports the MAE and R^2 for Matminer and DCF as a function of X_{T}, with error bars representing one standard deviation across seeds. The trends discussed in the main manuscript remain preserved within the corresponding uncertainty bands. The MAE

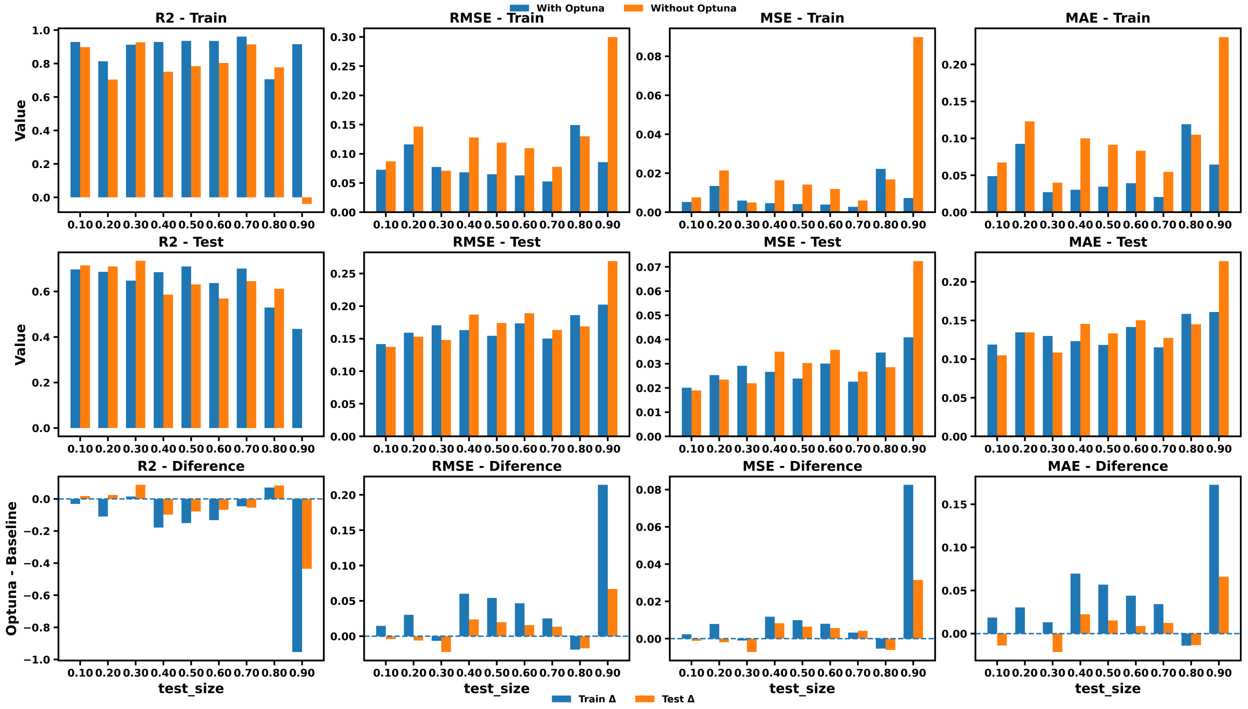

Figure S2: XGBoost performance with and without Optuna-based hyperparameter optimization for the DCF descriptor set. Top row, training values of R^2, RMSE, MSE, and MAE as a function of X_{T}. Middle row, corresponding test values. Bottom row, optimized minus baseline differences for training (blue) and test (orange).

shows a smooth and monotonic increase with X_{T} for both descriptor families, while R^2 remains essentially flat for moderate X_{T} and decays sharply at $X_{\mathrm{T}} = 0.9$, where the training set becomes too small for stable estimation. The size of the error bars grows with X_{T}, in agreement with the expected scaling of test set fluctuations for small training samples. The descriptor-level comparison between DCF and Matminer remains consistent under both repeated-split variability and hyperparameter optimization.

S4. Descriptor-level interpretability: graphene reference

The interpretability advantage that the main manuscript attributes to the DCF representation is intended at the level of the individual descriptor coordinates, that is, at the level of the variables that compose the input vector to the regression model. To illustrate this point

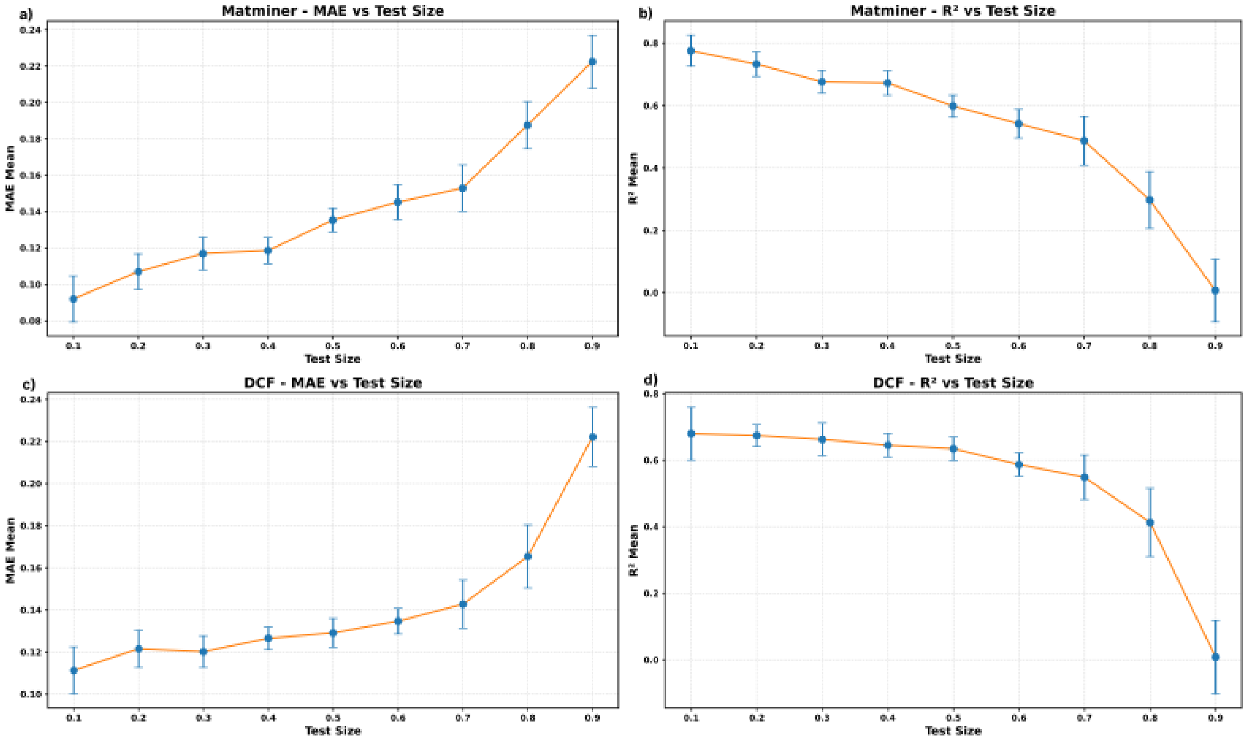

Figure S3: XGBoost results with statistical uncertainty across 20 random train and test partitions. Panels (a) and (b) show MAE and R^2 for the Matminer descriptor set. Panels (c) and (d) show MAE and R^2 for the DCF descriptor set. Error bars correspond to one standard deviation across seeds.

concretely, Fig. S4 compares the Matminer and the DCF descriptors computed for graphene, which corresponds to structure `34.cif` in the present dataset and which is well-characterized in the literature. The choice of graphene as a reference allows the descriptor values to be evaluated against a known structural baseline.

In the Matminer case, the representation consists of three scalar quantities from the density descriptor (panel (a)) and the discretized radial distribution function (panel (b). The scalar quantities admit a direct geometric reading, but the bulk of the representation is distributed across 200 distance bins of width 0.1 Å, each of which carries only a localized statistical weight associated with a narrow distance interval. While the radial distribution function as a whole reflects the structural ordering of graphene, the individual bin values are not, on their own, easily mapped to identifiable physical quantities such as transport regimes, orientational order, or lattice symmetry.

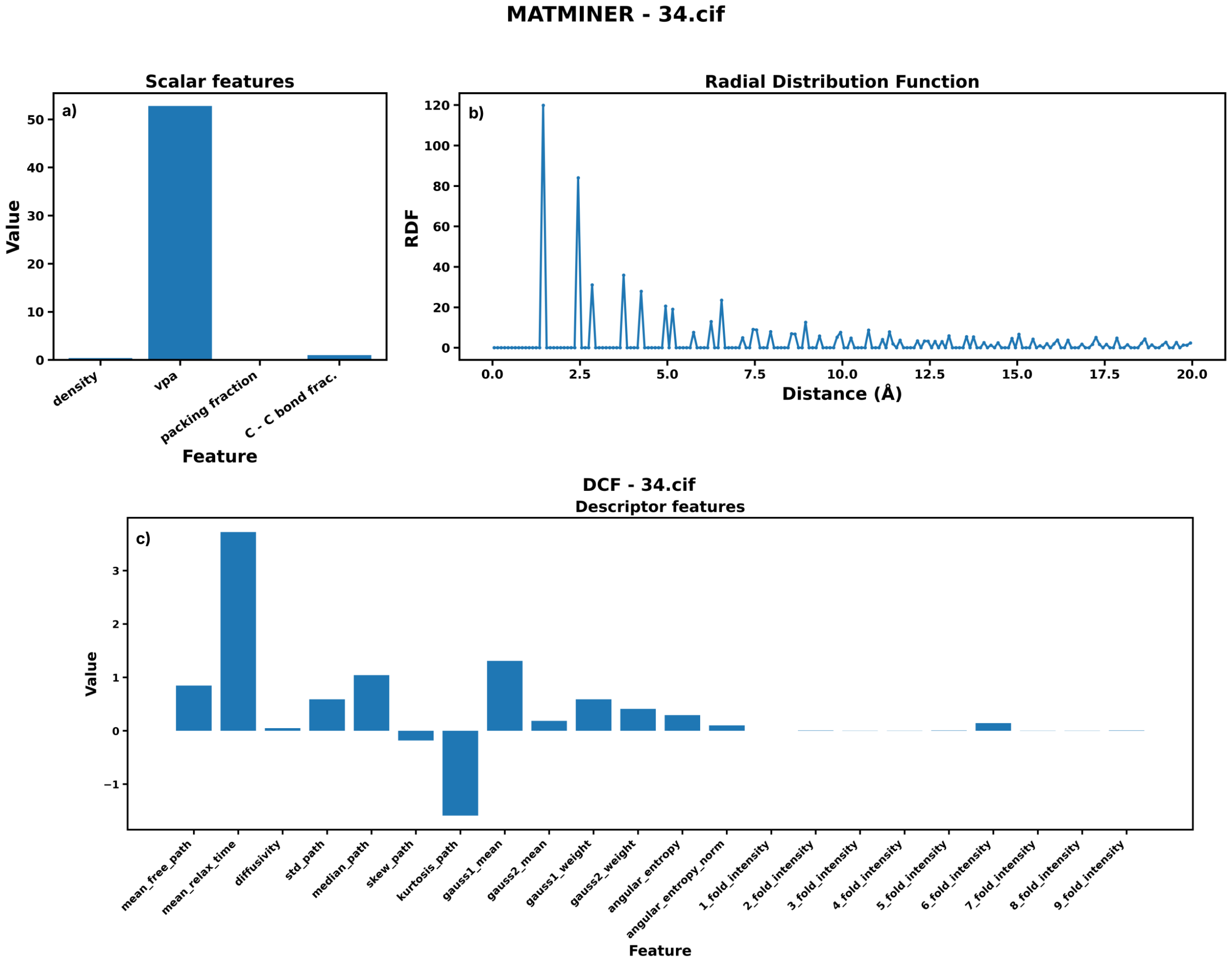

Figure S4: Descriptor-level comparison for graphene, structure `34.cif` in the present dataset. (a) Matminer density features (mass density, volume per atom, packing fraction). (b) Matminer radial distribution function (200 bins, cutoff 20.0 Å). (c) DCF features, including transport-related quantities, angular entropy, and rotational symmetry intensities from one-fold to nine-fold. The six-fold component in panel (c) corresponds directly to the hexagonal symmetry of graphene.

By contrast, the DCF representation shown in panel (c) is composed of a small set of variables, each of which carries an explicit physical meaning. The mean free path, the mean relaxation time, and the diffusivity quantify aggregated transport properties derived from elastic collision dynamics, while path-distribution statistics characterize the geometry of free-traversal events. The Shannon entropy of the angular distribution probes orientational order, and the Fourier intensities resolved into one-fold and nine-fold rotational symmetry directly probe the rotational character of the lattice. For graphene, the six-fold component

is the variable specifically designed to quantify hexagonal angular recurrence and is therefore expected to dominate over the other symmetry indices. The actual values of the descriptor coordinates are consistent with this expectation, providing a direct, descriptor-level link between the input representation and the well-established hexagonal symmetry of graphene. Analogous readings apply to other structures, in which different symmetry components and transport indicators dominate depending on the underlying lattice. The compact size of the DCF vector and the physical meaning of each coordinate together support the qualitative claim of interpretability made in the main manuscript.
